# Microsatellite instability and loss of heterozygosity detected in middle-aged patients with sporadic colon cancer: A retrospective study

**DOI:** 10.3892/ol.2013.1573

**Published:** 2013-09-12

**Authors:** NASIR KAMAT, MOHAMMED A. KHIDHIR, MOUIED M. ALASHARI, ULF RANNUG

**Affiliations:** 1 Department of Molecular Biosciences, The Wenner-Gren Institute (MBW), Stockholm University, Stockholm, Sweden; 2 Department of Genetic Research, Management of Natural Conservations, PO Box 64634, Al Ain; 3 Department of Pathology, Tawam Hospital, Abu Dhabi, United Arab Emirates

**Keywords:** colorectal cancer, microsatellite instability, mismatch repair defects

## Abstract

Microsatellite instability (MSI) is a mutator phenotype that results from a defective mismatch repair (MMR) pathway. The present study examined the incidence of MSI and loss of heterozygosity (LOH) according to five markers from the panel of the National Cancer Institute (NCI) in 38 colorectal cancer (CRC) patients from the United Arab Emirates (UAE). MSI and LOH were analyzed using fragment analyses in a multiplex PCR setting on a capillary array electrophoresis platform. The expression of the MMR proteins, hMLH1 and hMSH2, was analyzed using immunohistochemistry. The cohort consisted of 17 females (44.7%) and 21 males (55.3%) with mean ages of 59.9 and 63.3 years, respectively. The overall MSI incidence was 31.3% (95% CI, 16.1–50.0), and included three patients with high MSI (MSI-H; 9.4%; 95% CI, 2.0–25.0) and seven patients with low MSI (MSI-L; 21.9%; 95% CI, 10.7–39). LOH was detected in three patients, while the remaining 25 patients (65.8%) showed no instability and were therefore classified as microsatellite stable (MSS). MSI was detected in the following screened markers: Bat25 in seven patients, Bat26 in three patients, adenomatous polyposis coli (APC; D5S346) in five patients, AFM093xh3 (D2S123) in two patients and Mfd15 (D17S250) in three patients. Of the five MSI-positive patients, four (80%) were evidently younger, aged 38, 48, 49 and 59 years, respectively. The MSI-H incidence (9.4%) was lower compared with that of other ethnic groups. In terms of the MMR proteins, hMLH1 expression was deficient in seven patients, of whom three were MSI-H patients, and hMSH2 was deficient in three patients. Fisher’s exact test showed significant associations between hMLH1 and MSI when classified as MSS, MSI-L or MSI-H (P=0.0003). No such association was observed with abnormal MMR protein expression, age, cancer stage or gender.

## Introduction

Colorectal cancer (CRC) is a major health concern worldwide as one of the leading causes of cancer-associated mortality and the third most common cancer diagnosed in males and females (1,2). The global incidence rate of CRC is estimated to be 1.2 million new cases per year with a 50% mortality rate (3), and >90% of CRCs are adenocarcinomas that originate from benign adenomas of the lining layer of the bowel. Sporadic cases with no previous relevant history account for 70–75% of CRCs (4). CRCs of familial and sporadic origins are caused by a specific, but not necessarily similar, genetic instability (5,6). Increasing evidence is now available with regard to the significance of mutations that inactivate tumor suppressor genes, including adenomatous polyposis coli (APC), p53, PTEN and KRAS, in the tumorigenesis of colon cancer (7). A mutation in the APC gene is an early step in the tumorigenesis of colon cancer and is considered to be a hallmark of sporadic non-hereditary polyposis (6). The inactivation of tumor suppressor genes in colon cancer tumorigenesis is believed to be associated with invasiveness (8). The progression of genetic instability in colon cancer may follow various pathways, including chromosomal instability (CIN), which is detected in up to 80% of CRCs and may be accompanied by a loss of heterozygosity (LOH) and chromosomal rearrangement (9). Another pathway is marked by the occurrence of microsatellite instability (MSI), which represents a mutator phenotype that is observed in a number of CRC cases. MSI is believed to be caused by accumulated mutations in the CPG islands of hMLH1 and/or the hypermethylation of its promoter, which impairs the function of the key mismatch repair (MMR) protein (5,10). In brief, MSI is a change in the length of a repetitive DNA sequence in tumor tissues compared with the original length in the normal counterpart of the same patient (11). MSI occurs in the genome through a slippage process during DNA replication and causes frequent deletions/insertions of repeat units (12). Such replication errors may occur due to a decreased fidelity of the replication apparatus, which worsens if it is added to an impaired MMR system (12). Although germline and somatic mutations have been detected in various MMR genes, hMLH1 and hMSH2 have shown the strongest correlations with MSI in colon cancer (13). hMLH1 has been prevalently connected with sporadic and familial colon cancer pathogeneses (14). The main chemotherapy regime for advanced colon cancer cases is 5-fluorouracil, to which MSI colon cancers have shown resistance (15). Furthermore, MSI has been noted as an earlier step in the CRC progression pathway in >90% of hereditary non-polyposis colorectal cancer (HNPCC) patients and in 15–20% of sporadic cases (2,13). Similar forms of instability have been diagnosed in numerous other tumors (16,17). These findings highlight the higher tendency of MMR-deficient tissues to accumulate mutations, develop MSI and eventually progress to a cancerous state. Hence, it has been recommended that assessing the MSI status in CRC tumors is important in obtaining an improved diagnosis, interpretation and prediction of the disease outcome. In 1997, the National Cancer Institute (NCI) workshop developed the Bethesda guidelines (18), which recommend the use of a five-marker panel to assess the presence and extent of MSI in colon cancers. The NCI panel includes two mononucleotide repeat sequences (Bat-25 and Bat-26) and three dinucleotide repeat sequences (D2S123, D5S346 and D17S250). An MSI status determination is performed by comparing DNA from tumor and normal tissues. The results are classified according to the number of altered microsatellite sequences. If alterations are present in two or more of the five microsatellite sequences, the cancer is classified as high MSI (MSI-H). If only one marker is mutated, the cancer is classified as low MSI (MSI-L). If no changes are present among the five microsatellites, the tumor is considered to be microsatellite stable (MSS). Since the investigations of MSI and other instabilities in CRCs may not be sufficient to fully comprehend the instabilities and their causes, combining immunohistochemical techniques to assess MMR protein expression has also been recommended (13). The Evaluation of Genomic Applications in Practice and Prevention Working Group demonstrated that MSI detection is useful for diagnosing suspected HNPCC (19). In addition, several studies have suggested a prognostic value for MSI and LOH assessment (20,21). A number of regions around the globe, including the United Arab Emirates (UAE), lack adequate epidemiological data or active long-term cancer registries. Furthermore, the number of clinicopathological studies is scant. In 2008, a study was conducted at Howard University on archived colon cancers from the Sultanate of Oman to estimate the MSI status and MMR defects in this population (22). The study of the Omani subjects revealed an MSI incidence that was comparable with that of the United States. The present study analyzed the existence and extent of MSI, LOH and MMR deficiencies in archived CRCs from the Department of Pathology at Tawam Hospital (Abu Dhabi, UAE) between 2005 and 2008. This study is the second in the region and the first to investigate the frequency of MSI, LOH and MMR deficiencies in colon cancer patients in the UAE.

## Materials and methods

### Study design and tumor collection

Colorectal carcinomas specimens were archived between 2005 and 2008 by the Department of Pathology at Tawam Hospital. Approval for this study was obtained from the Al Ain Medical District Human Research Ethics Committee and the Faculty of Medicine and Health Sciences at UAE University (Al Muwaiji, Al Ain, UAE). Written informed consent was obtained from the patients. Specimens were collected in paraffin-embedded blocks for the genetic analyses of MSI and LOH and an immunohistochemical analysis of MMR protein expression. A total of 114 specimens from 38 CRC patients were analyzed. All specimens were screened against five microsatellite markers (NCI panel) and immunohistochemistry was performed for hMLH1 and hMSH2 expression, as they are the most frequently reported genes to harbor MMR defects in CRC patients. The sample of patients consisted of 21 (55.3%) males and 17 (44.7%) females, resulting in a male-to-female ratio of 12:10. The age and gender of the patients and the TNM classification of malignant tumors (TNM) are displayed in Table I.

### Genetic analysis

DNA extraction from the formalin-fixed and paraffin-embedded tissues was conducted using a QIAamp DNA FFPE Tissue kit from Qiagen Inc. (Valencia, CA, USA), which included a specific column to purify PCR-grade DNA from prefixed and paraffin-embedded tissues. However, in six patients (Table I, Patient nos. 33–38), no DNA was extracted or the quality of the extracted DNA was not suitable for the required PCR reactions. Hence, no microsatellite analysis was assessed for those patients.

Microsatellite analyses were performed using fluorescently labeled primers from Applied Biosystems (Carlsbad, CA, USA). These sequences have been described previously (23). Specific information with regard to the microsatellite marker panel, including the sequences that were used, is provided in Table II.

### PCR and fragment analysis

Single and multiplex PCR reactions were conducted using a Biometra T3000 Thermocycler (Biometra, Göttingen, Germany) to amplify the five markers recommended by NCI (Bat25, Bat26, D2S123, D5S346 and D17S250). The amplification reactions were carried out in a 20 μl reaction volume consisting of 1X Gold Amplitaq Master Mix (Applied Biosystems), with the addition of 100 ng purified genomic DNA and adjusted to a final primer concentration of 0.2 μM. The cycling conditions consisted of an initial denaturation cycle at 95°C for 5 min, followed by 29 cycles at 94°C for 1 min, 50, 55 or 58°C for 45 sec and 72°C for 50 sec, with a final 12-min extension at 70°C. A fragment analysis was conducted by loading the PCR products onto an ABI 3130 genetic analyzer (Applied Biosystems). The fragments were sized and compared using the Gene Mapper software (Version 4) from the same manufacturer. All runs included the use of an internal molecular weight control (LIZ 500 Genescan; Applied Biosystems).

Using capillary array electrophoresis, MSI may be demonstrated using two main features: *de novo* alleles that appear as new peaks (i.e., peaks that did not exist in the normal tissue genotype) and slipped pre-existing alleles for the few base pairs (23,24). However, a partial (>35%) to complete signal loss of one heterozygote allele is an indicator of LOH (25,26).

### MMR protein expression

Tissues were emeddded in paraffin and microdissected at a thickness of 4 μm for the MMR protein expression analysis. The TP-125 HLX Ultra Vision Plus Anti-Polyvalent HRP detection system (Lab Vision, Fremont, CA, USA) was applied using specific monoclonal antibodies for hMLH1 and hMSH2 (Cell Marque, Rocklin, CA, USA). Healthy tissue from each patient was used as an internal control, in addition to the standard controls that were provided by the manufacturer. The results were designated as positive when a ≥10% proportion of the cell nuclei were stained positively.

### Statistical analysis

Statistical analyses of categorical variables were performed using Fisher’s exact test, the Mann-Whitney U test and the Kruskal-Wallis test. Hypothesis testing for associations between MMR and MSI and for LOH was conducted using Fisher’s exact test. The correlations between gender and MMR, MSI and LOH, respectively, were also analyzed using Fisher’s exact test. When MSI was subdivided into MSS and MSI (MSI-L+MSI-H) categories, a Mann-Whitney U test was conducted to evaluate their associations with cancer stage and age, respectively. When the three original MSI categories (MSS, MSI-L and MSI-H) were kept, a Kruskal-Wallis test was conducted to evaluate their associations with cancer stage and age, respectively. A Mann-Whitney U test was used to evaluate the associations between cancer stage and MMR and LOH, respectively, and the associations between age and MMR and LOH, respectively. The exact two-sided P-values were calculated and P<0.05 was considered to indicate a statistically significant difference (27). All the results were calculated using SAS software (release 9.2; SAS Institute Inc., Cary, NC, USA).

## Results

### MSI and LOH

**Figure 1:**
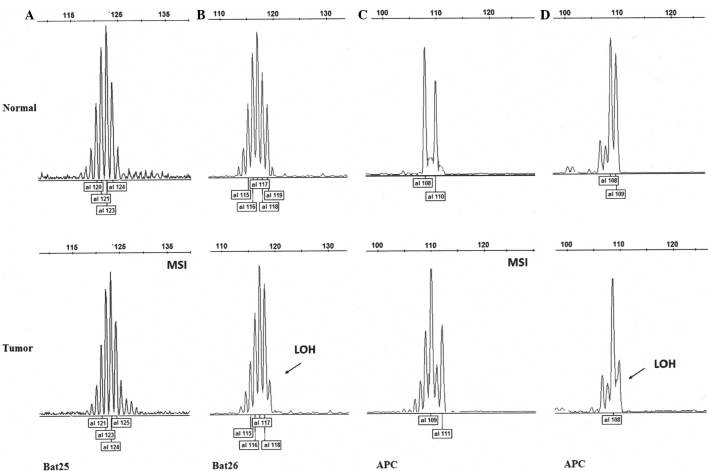
Microsatellite instability (MSI) and loss of heterozygosity (LOH) in three markers. The top panel shows the normal genotypes from the healthy tissues and the bottom panel depicts the instability (MSI and LOH) from the tumor tissues. (A) MSI at Bat25 from patient 4. (B) LOH at Bat26 from patient 21. (C) MSI at APC from patient 26. (D) LOH at APC from patient 30. APC, adenomatous polyposis coli.

MSI-H and -L-positive events were observed in 10 of 32 patients (31.3%), including three MSI-H patients (9.4%) and seven MSI-L patients (21.9%). LOH was detected in another three patients (9.4%), but the remaining 25 patients (78.1%) showed no instability and were classified as MSS. The frequencies of MSI and LOH at the individual marker levels are shown in Table I. Bat25 presented with MSI in seven patients (21.9 %) with no evidence of LOH. Bat26 showed MSI in three patients (9.4%) and LOH in one patient (3.1%). MSI at APC (D5S346) was evident in five patients (15.6%) and LOH was identified in two patients (6.3%; Fig. 1). Whereas AFM093xh3 (D2S123) and Mfd15 (D17S250) harbored MSI in two (6.3%) and three (9.4%) patients, respectively, there was no evidence of LOH events in the two loci.

### Age and gender

The same number of MSIs were detected in males and females (five of each gender), with a relatively higher incidence in females (29.4%) versus males (23.8%). Of the three MSI-H tumors, two were identified in females. Despite the relatively small study sample, four of the five male MSI patients were younger than their female counterparts (38, 48, 49, 59 and 69 years versus 64, 64, 65, 67 and 71 years, respectively; Table I).

### Tumor stage (TNM) and lymph node involvement

The tumor stages and MSI and LOH statuses are summarized in Table I. A large number of the sampled tumors were stage III (44.8%), while stage II and IV tumors were represented equally with 21% each. Stage I tumors accounted for 13.1% of the samples and six of the seven MSI-L tumors were at stage III and one was at stage II. Of the three MSI-H tumors, two were classed as stage IV and one as stage III. In total, two of the LOH tumors were at stage IV and one was at stage III. A pathological assessment of lymph node involvement estimated that tumor cells were evident in nine of the 10 MSI tumors (three MSI-H and six MSI-L).

### Expression of MMR proteins

**Figure 2:**
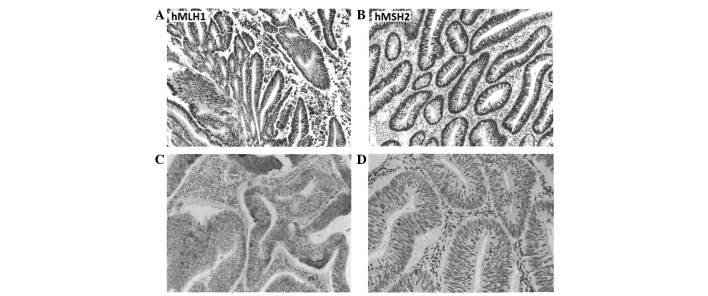
Immunohistochemical reactions for the MMR (mismatch repair) protein expression (x400). Positive and negative hMLH1 reactions from patients (A) 12 and (C) 14. Positive and negative hMSH2 results from patients (B) 9 and (D) 24.

An immunohistochemical analysis showed that the expression of the two studied MMR proteins, hMLH1 and hMSH2, was deficient in nine patients (23.7%), six of which were deficient in hMLH1, two in hMSH2 and one in both proteins (Table I). MSI-H events were evident in two of the six patients who showed hMLH1 deficiencies alone and in the single patient that was deficient in both MMR proteins. Fig. 2 shows examples of MMR protein expression in the patients.

### Statistical findings

Of the 38 patients in the studied population, 21 (55.3%) were male and 17 (44.7%) were female. The mean age of the population was 61.4 years and when subdivided by gender, the mean ages for the females and males were 63.3 and 59.9 years, respectively. The demographical and clinical characteristics are displayed in Table III.

### Incidence of MSI, LOH, and MMR

MSI was detected in 10 of 32 patients (31.3%; 95% CI, 16.1–50.0%). A total of seven of these 10 patients were classified as MSI-L and three were classified as MSI-H. LOH was detected in three of the 32 patients (9.4%; 95% CI, 2.0–25.0). MMR deficiencies, with regard to hMLH1, were detected in seven of 38 patients (18.4%; 95% CI, 7.7–34.3), and a loss of hMSH2 expression was detected in three of the 38 patients (7.9%; 95% CI, 1.7–21.4).

### Association tests

A lack of hMLH1 expression was identified in six of the patients accompanied with MSI, whereas only four of the 25 patients expressing hMLH1 showed MSI. A statistical analysis using Fisher’s exact test showed significant associations between hMLH1 and MSI when classified as MSS, MSI-L or MSI-H (P=0.0003), and between hMLH1 and MSI when subcategorized into MSS or MSI (MSI-L+MSI-H; P=0.0014). No association was observed between MSI and hMSH2, age, gender or cancer stage.

The tumors from the three patients showing LOH were classified as stage IV, and a statistical analysis using a Mann-Whitney U test revealed a significant association between LOH and the cancer stage (P=0.0079). No such association was observed for abnormal MMR protein expression, age, cancer stage or gender. The association tests are displayed in Table IV.

**Figure 3:**
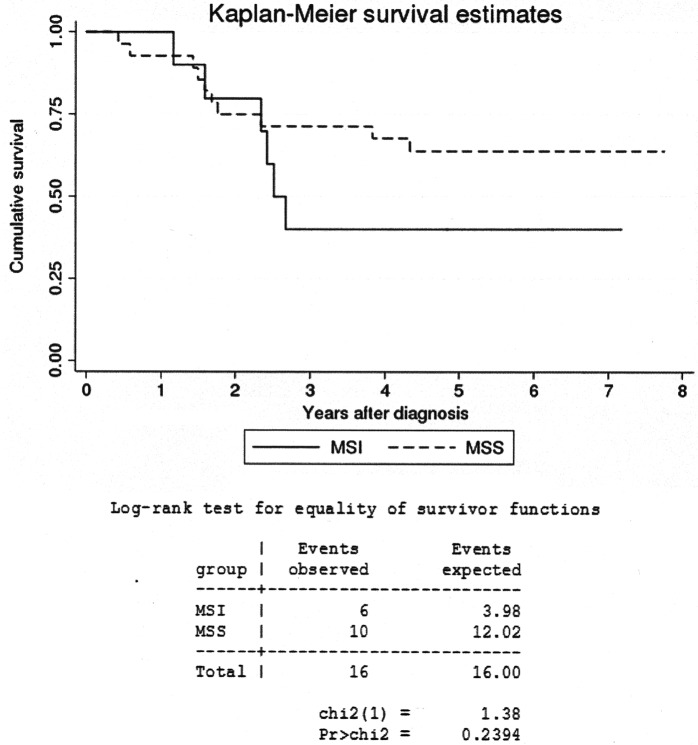
A Kaplan-Meier survival curve and log-rank test showing the difference in survival time between MSI-positive and MSS patients. MSI, microsatellite instability; MSS, microsatellite stable.

The length of patient survival following the diagnosis was averaged at 4.1 years. A Kaplan-Meier survival curve (Fig. 3) and a log-rank test revealed no significant difference in the survival times between the patients with MSI-positive tumors and patients with MSS tumors.

## Discussion

Genetic instability is a hallmark of most, if not all, known cancers. Genetic instability pathways have been shown to underlie the tumorigenesis of CRC. Of particular interest is MSI, which shows a marked variation in its incidence between hereditary and sporadic CRCs (85 and 15–20%, respectively) (2,13). Studies have elucidated the mechanisms that are believed to play a pivotal role in MSI occurrence to a certain extent, and a malfunctioning or inactivated MMR repair apparatus is considered to be a main cause (9,12). The resultant sequential accumulation of mutations and mismatches that accidently occurs during DNA replication is normally corrected by an intact MMR apparatus (12).

The present study is the first to be designed to reveal the incidence and clinical significance of MSI and LOH in patients with CRC in the UAE. An MSI incidence rate of 26.3% (7.9% MSI-H and 18.4% MSI-L) and an LOH rate of 7.9% were observed. An MSI-H incidence (MSI in two or more loci) was evident in 7.9% of CRC patients (three of 38). This rate is lower than the 12.2% that was detected in a previous study of another population (Omani patients) in this region (22). The difference was not statistically significant (Fisher’s exact test) and was most likely to be due to the relatively small size of the sample population. In line with the Omani study, MSI was detected in younger patients compared with studies of other populations (28,29), particularly among males (Table I). According to the available medical history, none of the studied patients had a family history of cancer predisposition. At least one female patient under our observation developed an ovarian tumor, which may indicate that she carried a germline mutation that may have predisposed her to HNPCC. MMR pathway inactivation is widely accepted as a mechanism for mediating MSI in colorectal carcinoma, particularly hMLH1 and hMSH2, which are the main two genes involved (6,30). In hereditary forms of CRC, it is well established that a mutation or epigenetic alteration, including hypermethylation of an hMLH1 promoter, are typical of this syndrome (9). Although hMLH1 and hMSH2 are implicated in sporadic CRC mutations, hMLH1 has been observed to be affected more frequently (31). In the present results, hMLH1 protein expression was deficient in seven patients (18.4%) and the hMSH2 protein expression was deficient in three patients (7.9%). The hMLH1 deficiency correlated with incidences of MSI-H and MSI-L. These results must be confirmed with a larger cohort of patients. LOH was detected in three patients (7.9%), including two at APC (5.3%) and one at Bat26 (2.6%). MMR protein expression was evidently intact in the three patients with LOH (Table I). The highest rate of MSI was detected in Bat25, in concordance with the reported data that mononucleotide markers are more sensitive to MSI than dinucleotide repeats (32–34). Of particular interest are the MSI and LOH events at APC, which were screened using the D5S346 marker. A total of five patients showed MSI and another two suffered LOH at this locus. Younger males, aged 48, 59 and 52, respectively, were identified to display two MSI and one LOH event at APC. The significance of these findings is emphasized by the frequently reported role of the inactivation of this tumor suppressor gene in CRC (35). In fact, it has been identified that APC is inactivated by LOH or epigenetic alteration (methylation) in several tumors, including CRC (36,37). In addition, in familial adenomatous polyposis (FAP), patients who inherit a germline mutation in the APC gene, the lifetime risk of developing CRC approaches 100% (38). It is worth noting that the same types of instability have been detected in hereditary and sporadic colon cancers, with varied rates and clinical significance (2). MSI and LOH statuses have been considered to be valuable and independent prognostic markers in CRC patients (20). Furthermore, the type of genetic instability inherited in patients with CRC may play a role in their survival time, as patients with HNPCC have a survival time that is estimated to be 5 years longer than that of patients with inherited FAP (39). Another significant link was identified between MSI and the response to certain chemotherapy agents. Colon cancers with MSI were noticeably resistant to 5-fluoruracil, which remains the treatment of choice in advanced CRC cases (15).

In terms of the correlation between tumor stage and lymph node involvement, of the 38 sampled tumors, 25 were in stages III or IV (16 in stage III and 9 in stage IV) and 13 were observed in stages I or II (5 in stage I and 8 in stage II). Stage IV tumors were detected in two of the three MSI-H cases (66.7%). Lymph node involvement was confirmed in nine of the 10 MSI tumors (90%), which may indicate why these tumors behaved more aggressively (Table I).

In conclusion, the incidence of MSI in CRC patients from the UAE is within the rates that have been reported in other studies of various ethnic and geographical backgrounds. The present patient group produced noteworthy molecular and clinical findings in terms of earlier ages of detection, heterozygosity losses and relations between MSI, lymph node involvement and tumor stage. Thus, these incidence rates and clinical findings must be verified through a larger study cohort or population-based study.

## Figures and Tables

**Table I:** CRC patients; basic information, TNM staging and results of MSI, LOH and MMR protein expression.

Patient no.	Age, years	Gender	Tumor stage, TMN	Stage	MSI detected in	LOH detected in	hMLH1	hMSH2
1	68	F	T3, N2, M1	4	MSS	APC	−	+
2	61	M	T2, N2, M0	3	MSS	No LOH	+	+
3	49	M	T3, N1, M0	3	MSS	No LOH	+	+
4	64	F	T3, N2, M0	3	Bat25	No LOH	−	+
5	67	M	T3, N1, M0	3	MSS	No LOH	+	+
6	70	F	T1, N0, M0	1	MSS	No LOH	+	+
7	48	M	T3, N2, M0	3	Bat25, Bat26, APC, Mfd15	No LOH	−	+
8	73	M	T3, N0, Mx	2	MSS	No LOH	+	+
9	65	F	T4, N2, M1	4	Bat25, APC, Mfd15, AFM093xh3	No LOH	−	−
10	69	M	T3, N1, M0	3	Bat26	No LOH	+	−
11	66	F	T3, N2, M1	4	MSS	No LOH	+	+
12	58	M	T3, N0, Mx	2	MSS	No LOH	+	+
13	72	M	T3, N0, M0	2	MSS	No LOH	+	+
14	59	F	T3, N2, M0	3	MSS	No LOH	+	+
15	38	M	T2, N1, M0	3	Bat25	No LOH	−	+
16	53	F	T3, N2, M1	4	MSS	No LOH	+	+
17	67	M	T2, N0, M0	1	MSS	No LOH	+	+
18	62	M	T3, N2, M1	4	MSS	Bat26	+	+
19	55	F	T2, N1, M0	3	MSS	No LOH	+	+
20	49	M	T3, N2, M0	3	Bat25	No LOH	+	+
21	71	F	T4, N2, M1	4	Bat25, Bat26, APC, Mfd15, AFM093xh3	No LOH	−	+
22	60	M	T2, N0, M0	1	MSS	No LOH	+	+
23	59	M	T3, N0, M0	2	APC	No LOH	+	+
24	68	F	T3, N0, Mx	2	MSS	No LOH	+	−
25	58	M	T3, N0, M0	2	MSS	No LOH	+	+
26	64	F	T3, N2, M0	3	APC	No LOH	−	+
27	67	M	T2, N1, M0	3	MSS	No LOH	+	+
28	60	M	T3, N1, M0	3	MSS	No LOH	+	+
29	59	F	T2, N0, M0	1	MSS	No LOH	+	+
30	52	M	T3, N2, M1	4	MSS	APC	+	+
31	67	F	T3, N1, M0	3	Bat25	No LOH	+	+
32	69	F	T2, N1, M0	3	MSS	No LOH	+	+
33	68	M	T2, N0, M0	1	N/A	N/A	+	+
34	59	F	T3, N0, M0	2	N/A	N/A	+	+
35	61	F	T3, N2, M1	4	N/A	N/A	+	+
36	57	M	T3, N1, M0	3	N/A	N/A	+	+
37	63	M	T3, N0, M0	2	N/A	N/A	+	+
38	58	F	T3, N1, M1	4	N/A	N/A	+	+

CRC, colorectal cancer; TNM, TNM classication of malignant tumors; MSI, microsatellite instability; LOH, loss of heterozygosity; MMR, mismatch repair; APC, adenomatous polyposis coli; M, male; F, female; N/A, not available.

**Table II:** Characteristics of microsatellite markers analyzed.

Name (locus)	Primer sequence, 5′→3′	Unit of repeats	PCR-Tm^c^	Dye	Size, bp
Bat-25					
F	TCG CCT CCA AGA ATG TAA GT				
R	TCT GCA TTT TAA CTA TGG CTC	1	55°C	NED	119–124
Bat-26					
F	TGA CTA CTT TTG ACT TCA GCC				
R	AAC CAT TCA ACA TTT TTA ACC C	1	55°C	FAM	112–127
Mfd15 (D17S250)					
F	GGA AGA ATC AAA TAG ACA AT				
R	GCT GGC CAT ATA TAT ATT TAA ACC	2	50°C	VIC	147–163
APC (D5S346)					
F	ACT CAC TCT AGT GAT AAA TCG				
R	AGC AGA TAA GAC AGT ATT ACT AGT T	2	55°C	FAM	107–131
AFM093xh3 (D2S123)					
F	AAA CAG GAT GCC TGC CTT TA				
R	GGA CTT TCC ACC TAT GGG AC	2	58°C	PET	209–232

F, forward sequence; R, reverse sequence; APC, adenomatous polyposis coli; Tm^c^, melting temperature.

**Table III:** Demographics and clinical characteristics of the studied population.

Characteristic	Subset where MSI and LOH were analyzed	All
Mean age ± SD, years	61.5±8.2	61.4±7.6
Gender, n		
Male	18	21
Female	14	17
Cancer stage, n		
I	4	5
II	6	8
III	15	16
IV	7	9
MMR, n		
hMLH1		
(+)	25	31
(−)	7	7
hMSH2		
(+)	29	35
(−)	3	3

MSI, microsatellite instability; LOH, loss of heterozygosity; MMR, mismatch repair.

**Table IV:** Correlations between MSI or LOH and hMLH1and hMSH2 and between these endpoints and age, gender and cancer stage, using different statistical methods.

Variables	Test of Association	P-value
MSI^a^, hMLH1	Fisher’s exact test	0.0003
MSI^a^, hMSH2	Fisher’s exact test	0.2238
MSI^a^, age	Kruskal-Wallis test	0.7907
MSI^a^, gender	Fisher’s exact test	0.8560
MSI^a^, cancer stage	Kruskal-Wallis test	0.1865
MSI^b^, hMLH1	Fisher’s exact test	0.0014
MSI^b^, hMSH2	Fisher’s exact test	0.2238
MSI^b^, age	Mann-Whitney U test	0.5954
MSI^b^, gender	Fisher’s exact test	0.7120
MSI^b^, cancer stage	Mann-Whitney U test	0.2576
LOH, hMLH1	Fisher’s exact test	0.5363
LOH, hMSH2	Fisher’s exact test	1.0000
LOH, age	Mann-Whitney U test	0.8615
LOH, gender	Fisher’s exact test	1.0000
LOH, cancer stage	Mann-Whitney U test	0.0079
hMLH1, age	Mann-Whitney U test	0.9342
hMLH1, gender	Fisher’s exact test	0.2074
hMLH1, cancer stage	Mann-Whitney U test	0.0537
hMSH2, age	Mann-Whitney U test	0.1127
hMSH2, gender	Fisher’s exact test	0.5768
hMSH2, cancer stage	Mann-Whitney U test	0.7046

a Microsatellite instability (MSI): microsatellite stable (MSS), low MSL (MSI-L), high MSL (MSI-H).

b MSI: MSS, (MSI-L+MSI-H).
